# Immunolocalization of anti-Mullerian hormone in bovine testes and ovaries during fetal development

**DOI:** 10.1590/1984-3143-AR2025-0088

**Published:** 2026-06-15

**Authors:** Vanessa Cunha Brito, Gabriela Santos Cruz, Moisés Moreira Lima, Eduardo Baia de Souza, Anelise de Sarges Ramos, Maria Auxiliadora Pantoja Ferreira, Moysés dos Santos Miranda, Nathália Nogueira da Costa de Almeida, Marcela da Silva Cordeiro, Simone do Socorro Damasceno Santos

**Affiliations:** 1 Laboratório de Fertilização In Vitro Prof. Dr. Otávio M. Ohashi, Instituto de Ciências Biológicas, Universidade Federal do Pará, Belém, PA, Brasil; 2 Laboratório de Biologia do Desenvolvimento e Imunohistoquímica, Instituto de Ciências Biológicas, Universidade Federal do Pará, Belém, PA, Brasil; 3 Laboratório de Células Tronco em Medicina Veterinária – LABTRON, Universidade Federal do Pará, Castanhal, PA, Brasil; 4 Instituto Federal de Educação, Ciências e Tecnologia do Pará, Ananindeua, PA, Brasil

**Keywords:** AMH, immunohistochemistry, testis, ovary, bovine fetus

## Abstract

The anti-Mullerian hormone (AMH) or Mullerian Inhibiting Substance (MIS), produced by the Sertoli cells induces the regression of Mullerian ducts in male embryos. Granulosa cells from ovarian follicle are homologous to Sertoli cells and produce AMH. The aim of this study was the immunolocalization of AMH in the testicles and ovaries of bovine fetuses at different ages. Gonads from bovine fetuses between 4 and 7 months (24-98 CRL) were collected in a local slaughterhouse, fixed in 10% formaldehyde, and processed for conventional histology and embedded in paraffin cut with a rotative microtome, deparaffinized, and subjected to immunohistochemistry using the anti- AMH 1:50 (SC 28912, Santa Cruz Biotechnology) according to the manufacturer's instructions. Immunostaining was observed in the cytoplasm of Sertoli cells, within the sexual cords (developing seminiferous tubules) at all ages and was not observed in gonocytes and interstitial tissue. In the ovary there was light staining in granulosa cells but not in the theca cells, however, intense staining was observed in the cytoplasm of the oocyte of primordial, primary, secondary and antral follicles, at all ages analyzed, suggesting that despite being produced by granulosa cells, AMH concentrates in the oocyte cytoplasm. The importance of AMH in sexual differentiation and action in Muller's ducts is well understood, however there are no studies on the role of this protein in gonadal development, this being the first report of AMH immunolocalization in fetal bovine testis and ovaries, requiring further studies on the action and importance of AMH in the gonadal development of bovine fetuses.

## Introduction

Anti-Müllerian Hormone (AMH) is a protein molecule, belonging to the Transforming Growth Factor beta superfamily (TGF-β), along with other substances such as inhibin, activin and GDF-9 that act during gonadal development and present effects stimulants or inhibitors in the division and differentiation of germ cells, in such a way that they help in the regulation of fertility (Josso and Di Clemente, 1999: Rey et al., 2003) and while most members of this superfamily have several functions, the action of AMH is restricted to the reproductive organs (Lee and Donahoe, 1993), however, its action and receptors have been observed more recently in other organs (Wang et al., 2005; Clarkson et al., 2011; Silva and Giacobini, 2021; Gowkielewicz et al., 2024). It is a homodimeric disulfide-linked glycoprotein with a molecular weight of 140 kD, strongly expressed in Sertoli cells, more specifically in the rough and Golgi complex endoplasmic reticulum (Tran and Josso, 1982; Hayashi et al., 1984), since the testicular differentiation until puberty and to a much lesser degree in granulosa cells from birth to menopause (Josso et al., 2001) and both Sertoli and granulosa cells express the AMH type II receptor (Di Clemente et al., 1994; Baarends et al., 1995), suggesting that AMH has an autocrine role, being inhibitory of the proliferation of Leydig cells (Behringer et al., 1994; Bergadá et al., 2006 [Bibr B013]), and in ovaries of mice function as a regulator of the initial and cyclic recruitment of ovarian follicles (Durlinger et al., 2002).

In rats, the Müllerian ducts are sensitive to AMH on the 14th day of gestation, and regression occurs between the 16th and 20th day of gestation, being insensitive after this period (Picon, 1969). AMH expression by Sertoli cells remains at high levels during fetal and prepubertal life then decreases to levels like ovarian levels in contrast to granulosa cells which maintain a low and constant level of AMH expression throughout life reproductive system (Josso et al., 2001).

The expression of AMH by Sertoli cells is regulated by androgens, which are the most important (Rey et al., 1994; Boukari et al., 2009); by gonadotropins that stimulate AMH expression by Sertoli cells, (Rey et al., 1993; Aksglaede et al., 2010) and by germ cell maturation which has an effect on AMH expression as it was observed that Sertoli cells from tubules where germ cells have started meiosis stop producing AMH earlier than those in which germ cells have not yet entered meiosis (Al-Attar et al., 1997).

In adult male mice it was shown that high levels of AMH affect the development of the reproductive system causing cryptorchidism, underdeveloped epididymis and absence of seminal vesicles and high levels of AMH after birth can interfere with the regulation of steroidogenesis of Leydig cells affecting development and maintenance of spermatogenesis (Behringer et al., 1990; Lyet et al., 1995).

In cattle, the freemartin sexual abnormality causes infertility in females born from twin-to-male births because the fetuses divide the placental membranes and fluids, with blood and hormone exchange, changing the characteristics of the female fetus, with the AMH being responsible for the gonadal changes observed in this condition (Vigier et al., 1984).

Granulosa cells, homologous to Sertoli cells, also produce AMH, but expression begins in the perinatal period, being restricted to granulosa cells on the sixth day after birth (Vigier et al., 1984). Granulosa cells from primary and early antral follicles show AMH expression and in antral follicles only granulosa cells close to the oocyte and some surrounding the antrum produce, however, atretic follicles and theca cells do not express (Münsterberg and Lovell-Badge, 1991). Costa et al. (2025) observed AMH immunostaining in granulosa cells and Sertoli cells of ovarian and dorsal testers of buffalo fetuses at 5-8 months.

In humans, AMH blocks meiosis in the fetal ovary leading to loss of germ cells and inhibits aromatase and LH receptors (Josso et al., 1998) observed in granulosa cells from preantral follicles of fetal ovaries only from week 36 (Rajpert-De Meyts et al., 1999). In sheep, it was detected in pre- and post-birth granulosa cells (Bézard et al., 1987) and Durlinger et al. (1999) mention that females without AMH present early follicular depletion, with its expression in human females GC induced by BMP -2, -6, -7 and -15 (Ogura-Nose et al., 2012).

Adult human ovaries show AMH restricted to granulosa cells from primary, secondary, preantral and antral follicles and GC from primordial follicles, oocyte and ovarian stroma show no marking for AMH (Weenen et al., 2004) however, the presence of AMH has been observed in oocytes, ovarian stroma, and theca cells along the granulosa layer of growing follicles (Stubbs et al., 2005).

In ovaries of rodent fetuses, AMH is not detected (Ueno et al., 1989; Durlinger et al., 2001) and in ovaries of human fetuses AMH expression occurs in granulosa cells around the 13th week and continues throughout the week of follicular formation where GC envelops the oocyte to form primordial follicles, suggesting that AMH may play a role during fetal ovarian development and these differences in AMH expression pattern between species and this contrast with that observed in humans make it difficult to understand the role of AMH in ovarian physiology (Modi et al., 2006).

AMH inhibits the growth of primordial follicles and by immunohistochemistry it was observed that AMH is located in ovarian stromal cells that surround the nests of germ cells and neo-formed primordial follicles and that in ovaries of adult animals AMH acts by inhibiting the transition of primordial to primary and is produced by granulosa cells of secondary and early antrum to antral follicles formation, and the role of AMH in neonatal and adult ovaries is to inhibit the progression of the primordial follicle (Nilsson et al., 2011) and in prepubertal and adult pigs, in immunohistochemical studies, the expression of AMH was observed in the granulosa cells of primordial follicles, primary follicles, preantral and preovulatory and in the oocyte (Almeida et al., 2012).

AMH is one of the most important factors in controlling the rate of activation of primordial follicles and, like BMP's, also members of the TGFβ superfamily, AMH plays a key role in suppressing the recruitment of primordial follicles by reducing SCF expression (Stem Cell Factor), also known as Kit Ligand, a cytokine produced by the granulosa cells of primordial follicles, and Fibroblast Growth Factor β (FGFβ) produced by the oocyte that appears to stimulate the transition from primordial to primary in several species (Dunlop and Anderson, 2015) and inhibits VEGF production by granulosa cells and GDF-9 in human ovaries by modulating follicular vascularization on which follicular maturation depends (Detti et al., 2015). In mice, it inhibits the effect of FSH on follicular growth in vitro, negatively affecting the expression of a large number of genes involved in folliculogenesis and decreasing estradiol levels, reducing follicular atresia, aromatase expression and intracellular cAMP levels (Hayes et al., 2016).

AMH inhibits GC proliferation in in vitro culture and oocyte-produced factors such as BMP-15 increase the expression of AMH, AMH-R2 and transition factor WT1 in GC from 5 to 8mm follicles in cattle and this increase may initiate AMH signaling that can suppress the expression of CYP19A1 (responsible for the production of aromatase that converts androgens into estrogen) and the proliferation of GC, and together these actions keep the follicles in a primordial stage (Poole et al., 2016) and regulates estrogen levels through its ability to modulate ovarian sensitivity to FSH (Jurczak et al., 2016).

Mammals bi-directionally differentiate their reproductive organs during early embryonic development and the Müllerian ducts give rise to the upper third of the vagina, the cervix, the body of the uterus, uterine tubes and the ovarian tunica albuginea (Renaud et al., 2005) and the Wolffian ducts differentiate into seminal vesicles, the vas deferens and the epididymis (Goodfellow and Lovell-Badge, 1993) in female and male embryos, respectively. The genesis of one set of ducts, along with the atrophy of the other, is controlled by hormones AMH and testosterone. In female embryos, the lack of testosterone is responsible for the atrophy of the Wolffian ducts. In male embryos, the presence of AMH results in the regression of structures derived from the Müllerian ducts (Goodfellow and Lovell-Badge, 1993). AMH also affects other types of cells in fetal life, for example, it can have disadvantageous effects on the maturation of type II pneumocytes, which explains the more frequent occurrence of respiratory distress syndrome in prematurely born male infants (Catlin et al., 1990).

AMH is synthesized both in the testes and the ovaries but with a different timing. During development of the male fetuses, AMH is expressed in the Sertoli cells of gonads as early as 8 weeks of pregnancy (48 days post conception) (Rajpert-De Meyts et al., 1999; Mamsen et al., 2017; Lucas-Herald and Mitchell, 2022), and then it reaches high serum values (~50ng/mL) (MacLaughlin and Donahoe, 2010; Kim et al., 2014). In post-natal males, the AMH level is inversely proportional to the testosterone level (Teixeira et al., 1999).

In female fetuses, AMH gene expression starts in GCs of preantral ovarian follicles at 36 weeks of gestation (Rajpert-De Meyts et al., 1999) and AMH proteins are not detected in the serum until the 37^th^ week of gestation (Guibourdenche et al., 2003). In contrast, a more recent study indicated that AMH gene expression on GCs started from 23 weeks of gestation and serum AMH levels are detectable in female newborns born after 25 weeks and 6 days of pregnancy (Kuiri-Hänninen et al., 2011).

AMH being secreted by preantral and small antral follicles (up to 8 mm in diameter) as a paracrine factor inhibits the transition of primordial follicles into the primary follicle (Dewailly et al., 2014; Kumariya et al., 2021).

The effect of AMH is opposite to that of other members of the TGFb family, such as GDF and BMPs, which promote primordial follicles transition into primary follicle. Phosphorylation of the transcription factor FOXO3 (forkhead box O3) promotes the conversion of primordial follicles into primary follicle, while AMH inhibits FOXO3 phosphorylation and induces autophagy in the oocyte (Castro et al., 2016; Sonigo et al., 2019) and the chronic expression of AMH in early stages of female fetal development may exercise a harmful effect on primordial follicles leading to apoptosis via autophagy (Kumariya et al., 2021)

There seems to be a feedback loop between the gonads and the pituitary gland, in which AMH increases follicle-stimulating hormone (FSH) and luteinizing hormone (LH) synthesis in the pituitary gland, while FSH reduces the production of AMH in the ovaries (Bédécarrats et al., 2003; Grynberg et al., 2012; Devillers et al., 2019) and increases in the testes (Josso and Picard, 2022; Lucas-Herald and Mitchell, 2022). In males FSH positively controls AMH secretion by activating the AMH promoter through phosphorylation of different transcription factors, like SF1 (steroidogenic factor 1) and SOX9 (Josso and Picard, 2022; Lasala et al., 2011; Josso and Rey, 2020)

AMH appears to play an important role in the pathogenesis of PCOS which is characterized by chronic anovulation, hyperandrogenism and distinctive ovarian morphology (Pellatt et al., 2011; Norman et al., 2007). In women with PCOS, the plasma concentration of AMH is 2-3 times higher (Wang et al., 2017) or even 12 times more than the norm (Tal et al., 2014). Hence, the concentration of AMH inside the ovarian follicle is higher in patients with PCOS (Pellatt et al., 2007).

It was confirmed that AMHR2 is expressed in hepato-carcinomas (HCC), colorectal (CRC), non-small-cell lung (NSCLC) and renal cancer cells (RCC) and was also detected in melanoma and head and neck cancer cells (Barret et al., 2021).

For a long time, it was recognized that AMH did not cross the blood-brain barrier, and its action at the brain level was linked to the paracrine secretion of neurons, however, recent studies showed a point of contact at the level of the median eminence, outside the blood-brain barrier, where the GnRH afferents of neurons can come into contact with the circulating AMH (Weenen et al., 2004). It has been demonstrated in mice that the number of motoneurons and cerebella Purkinje cells were sex-dependent and that AMH could be involved in this phenomenon.

In 13-15-day-old mouse fetuses and 5-8-week-old adult mice showed that AMH determines physiological densification of neurons during fetal life, in adult mice, AMH ensures proper autocrine and paracrine functions, especially in the case of neuron damage (Chang et al., 2000; Wang et al., 2005) and in motor neuron disorders AMH has a protective effect and could be therapeutically advantageous being the gene expression of AMH is highest in in motor neurons with levels comparable to that in Sertoli cells and ovarian GCs and the expression strength of AMHR2 is 30 times higher in motor neurons than in other parts of the brain, the spinal cord or muscles (Wang et al., 2005).

In mice, Wang et al. (2005) suggests that MIS is a regulator of mature motor neurons. Motor neurons produce MISRII, which is the unique receptor for MIS, as well as the three types I receptors that associate with MISRII. Furthermore, MIS supported the survival and differentiation of embryonic motor neurons*in vitro*, indicating that activation of MIS receptors leads to downstream functional consequences in motor neurons and in immature motor neurons, also observed in vitro an action of AMH on neuronal differentiation and survival (Clarkson et al., 2011).

AMH appears to play a role in brain development (Wang et al., 2009) and sexual dimorphism has been demonstrated in mouse models in the neuronal composition of the bed of the nucleus of the stria terminalis (BNST) (Wittmann and McLennan, 2013). Male mice have a higher number of BNST neurons than female mice, with males having a higher number of neurons in mice expressing AMH compared to the mutated model for this gene. Mice deficient in AMH or its receptor would also show discreet signs of feminization of their spinal motor neurons and their exploration behaviors.

In mice, GnRH neurons themselves have been shown to produce AMH from embryonic life and GnRH neurons also express AMH in human fetus (Silva and Giacobini, 2021) and AMHR2 is expressed in both GnRH neurons in mice and human fetuses (Cimino et al., 2016). Neurons also synthesize AMH suggesting that it acts centrally to control the activity of neurons that produce GnRH and with the finding that AMH only regulates FSH and that this regulation is dimorphic between immature male and female rats it is suggested that AMH plays a key role in the hormonal complex that precedes puberty in females, which is a key factor in male sexual differentiation and regulates pituitary gonadotropic activity, giving new perspectives on understanding reproduction control, and although AMH receptivity has been identified in the brain and pituitary, the ability of the AMH to interfere with the hypothalamic-pituitary control of reproduction still needs clarification (Garrel et al., 2016).

AMH is considered a factor regulating the synaptic transmission in the hippocampus and with its paracrine/autocrine function it may influence processes, such as learning and memory, and functions as a protective and growth factor, and may support the researchs and treatment of Alzheimer’s disease (Wang et al., 2020).

The presence of AMHRII receptors in the human heart, and in the heart and aorta of mouse models, supports the hypothesis that AMH may also play a role in cardiovascular disease. AMHRII receptor interacts with the BMP protein pathway, whose role in angiogenesis and vascular maintenance and, due to its ability to inhibit the cell cycle and induce apoptosis, it has also garnered interest in oncology, with antibodies targeting AMHR2 are being investigated for their potential in diagnosing and treating various cancers (Gowkielewicz et al., 2024).

Some studies on gonadal development in bovine fetuses were carried out by several authors in female (Monteiro et al., 2003; Diniz et al., 2005; Antonino et al., 2017; ) and male fetuses (Souza et al., 2017) evaluating the morphological development, follicular population, testicular development and testosterone concentration, however, there are no studies on the presence of AMH during gonadal development in male and female bovine fetuses, which is the objective of this work.

## Methods

Fetal gonads were collected from animals that had already been slaughtered at the SOCIPE slaughterhouse (Belém, Pará, Brazil), thus eliminating the need for approval by the university's research ethics committee.

Testicles and ovaries of bovine fetuses were collected in a slaughterhouse and processed at the Laboratory of Histological Techniques at the Federal University of Pará. Fetal age was determined by the craniocaudal length (CRL), and the gonads were fixed in 10% formalin for 24h, dehydrated in dedincreasing concentrations of alcohol, diaphanized in xylene, infiltrated and embedded in paraffin and 5µm cuts were made in a rotating microtome. A slide from the gonads of each animal was stained with H.E to analyze the efficiency of histological processing.

### AMH immunolocalization

For AMH immunolocalization, the slides with histological cut of the testes and ovaries were deparaffinized in xylene, rehydrated in ethanol, washed in buffered saline (PBS) and incubated in 3% hydrogen peroxide in methanol for 30 minutes, immersed in citrate buffer of sodium heated at 70°C for 25 minutes and blocked with 10% normal goat serum (16210072, Invitrogen, Burlington, ONT, Canada) for 1 hour and incubated "overnigth" with primary anti-AMH antibody (MIS - H- 300) (SC 28912) at a dilution of 1:200 for 12 hours.

After that, they were washed in 0.05% tween PBS (TWEEN 20, 25564, EMS, Hatfield, PA.) and post-incubated in peroxidase-conjugated anti-rabbit IgG secondary antibody (SC 2030) diluted 1:200 for 2 hours. The reaction was developed in a solution of DAB (3,3' diaminobenzidine) (750118, Invitrogen, Burlington, ONT, Canada) for 5 minutes and then washed in distilled water, stained with HE, dehydrated and mounted with Entelan (Merck) and coverslip. For the negative control, PBS was used instead of the primary antibody. This entire procedure was in accordance with the manufacturers' instruction.

### Microscopic analysis

The AMH immunolocalization analyzes were performed in an Eclipse Ci-E photomicroscope (Nikon Corporation, Tokyo, Japan) coupled to a NIKON DS-Ri1 digital camera (Nikon Corporation, Tokyo, Japan) and NIS-Elements Basic Research software - NIKON Version 4.0.

## Results

The results show immunolocalization of AMH in testis and ovaries of bovine fetus at different ages described below.

### Testis

Thirteen pairs of testes were collected from bovine fetuses aged 4 to 7 months (24-79cm CRL) according to Table 1.

**Table 1:** Distribution of male bovine fetuses collected according to age determined by CRL and immunolocalization of AMH.

**Fetal age**	**Fetus number (n)**	**AMH Immunolocalization**		
		Sertoli Cell Gonocites Interstice		
4 months	4	+	_	_
5 months	5	+	_	_
6 months	2	+	_	_
7 months	2	+	_	_

+ positive; - negative.

**Figure 1:**
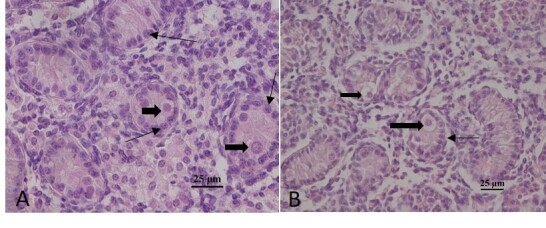
Histological aspects of bovine fetal testis at 4 (A) and 6 (B) months showing Sertoli Cells (SC ) and Gonocytes (G ).

Staining with H.E showed sexual cords (developing seminiferous tubules) well preserved with the presence of a tubular lining epithelium formed by Sertoli cells and the presence of few gonocytes (Figure 1).

**Figure 2:**
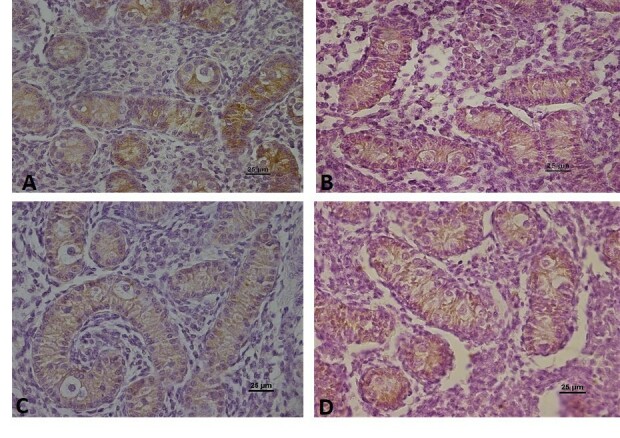
Immunolocalization of Anti-Mullerian Hormone (AMH) in the testes of bovine fetuses at 4 months (A), 5 months (B), 6 months (C), and 7 months (D) of gestational ages.

AMH immunostaining was observed in the cytoplasm of Sertoli cells, within the sexual cords at all ages analyzed, and the presence of AMH was not observed inside the gonocytes and interstitial tissue at any age analyzed (Figure 2).

### Ovaries

Ovaries from bovine fetuses between 4 and 7 months of age were collected as shown in the Table 2 below.

**Table 2:** Distribution of female bovine fetuses collected according to age determined by CRL.

Fetal age	Fetus number	AMH Immunolocalization	
		Granulosa cells Oocyte	
4 months	3	+	+
5 months	2	+	+
6 months	2	+	+
7 months	4	+	+

+ positive.

### AMH immunolocalization

**Figure 3:**
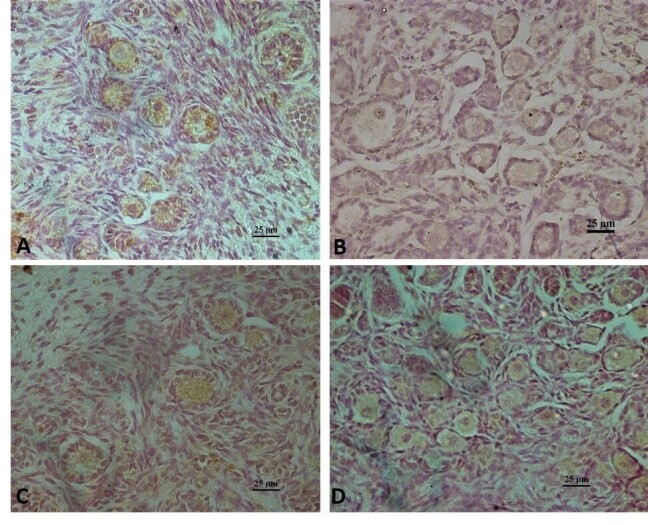
Immunolocalization of Anti-Mullerian Hormone (AMH) in the ovaries of bovine fetuses at 4 months (A), 5 months (B), 6 months (C), and 7 months (D) of gestational ages.

**Figure 4:**
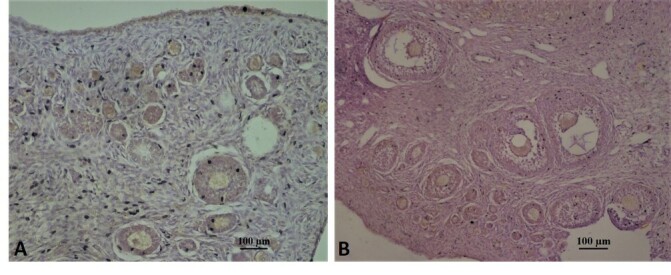
AMH immunolocalization in ovaries of 2 bovine fetuses (A and B animals) at 7 months were is observed secundary and antral follicles with immunolocalization in the oocyte citoplasm.

**Figure 5:**
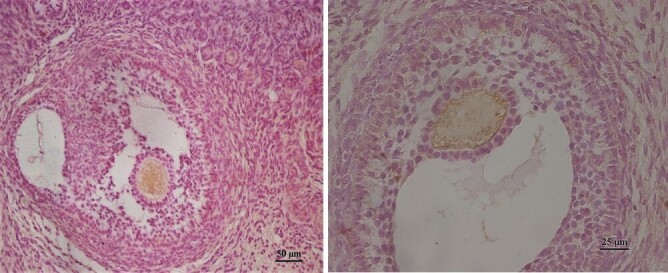
Antral follicles of bovine fetus at 7 months showing the immunolocalization of AMH in the oocyte citoplasm, and a light immunoreaction in the granulosa cells, primordial follicles and stroma.

AMH immunostaining was observed between 4 and 7 months in primordial and primary follicles, the location being specifically stronger in the cytoplasm of oocytes (Figure 3). Secondary and antral follicles were observed at 7 months of age, with strong AMH labeling in the oocyte cytoplasm and light labeling in the granulosa cells of these follicles (Figures 4 and 5).

## Discussion

### Immunolocalization of AMH in fetal bovine testes

Considering that the role of AMH in gonadal development is not well understood, this is the first report of the immunolocalization of AMH in fetal bovine testis showing the production of AMH during gonadal development in the male fetus.

The marking of AMH in the testes at different fetal ages demonstrates that testicular development, as well as sexual differentiation, is influenced by the action of this hormone, and this continuous expression observed throughout development demonstrates the activity of Sertoli cells leading us to infer that some type of interaction between these two cell types occurs, such as in the regulation of receptors and/or cell proliferation. The presence of AMH did not occur in the interstitial space where the Leydig cells responsible to produce testosterone are found.

Indeed, SRY induces the expression of the AMH gene via the SOX 9, SF1, WT1, DAX1, GATA4 genes which will directly regulate the expression of AMH in the fetal testis. the temporal expression of key regulatory genes governing sex-differentiation showed that in testis aged 48—64 days, AMH was highly expressed whereas in ovary no expression was detected. In contrast, there was higher expression of AMHR in ovary than testis (Mamsen et al., 2017) and the induction of AMH secretion will thereafter be dependent on FSH regulation.

It is the binding of AMH type II receptor in the mesenchymal cells of the Müllerian ducts that induces the morphological changes leading to degeneration of the Müllerian duct system. It may be related to an apoptotic mechanism of epithelial cells associated with changes in the basal membrane in response to paracrine signals (Mullen and Behringer, 2014). AMH is one of the earliest testis-specific proteins expressed by the embryonic male gonad from the 8th week of amenorrhea and provokes irreversible Müllerian duct regression, which is completed by the end of week 9 (Rey et al., 1996).

AMH rises quickly after birth, with a peak at three to six months, then the level stabilizes and lasts until puberty, establishment of a negative feedback control on the secretion of AMH by intratesticular testosterone, which level increases sharply at puberty (Boukari et al., 2009). At puberty, the polarity of Sertoli cells changes with the formation of the blood-testis barrier and the development of tight junctions between Sertoli cells: the secretion of AMH then becomes luminal and no longer basal. A plasma concentration is then obtained which decreases in favor of that of the seminal fluid (Holst et al., 2020).

Estradiol upregulates AMH expression by increasing the activity of the human AMH promoter in the prepubertal Sertoli cell line SMAT1 by signaling through Estrogen Receptor ER, which binds to a specific ERE (Estrogen Response Element) sequence (Valeri et al., 2020). Persistant Müllerian duct syndrome (PMDS), an autosomal recessive genetic syndrome occurring in 46, XY individuals, is the consequence of mutations in the AMH gene (PMDS type I) located on chromosome 19 (19p13) or in the gene for its type II receptor located at 12q13 (PMDS type II) and these patients present testes but most often in an intra-abdominal ectopic position because the testicular descent is AMH-dependent (Xu et al., 2019).

The presence of AMH observed in this work corroborates the work of Ball et al. (2008) who observed, using the immunofluorescence technique, the presence of AMH in the testes of equine fetuses at 5.5; 10 and 11 months of gestational age, indicating that AMH expression by Sertoli cells remains at high levels during fetal development.

Our results were like what was observed in other mammalian species, showing the presence of AMH in Sertoli cells during gonadal development (Behringer, 1995; Lee et al., 2003; Costa et al., 2025). Sertoli cells produce one or more factors that act on Leydig cells during fetal life, which are AMH and Desert hedgehog (Dhh) (Migrenne et al., 2012). AMH remains elevated until puberty, decreasing through the maturation of Sertoli cells (Josso et al., 1993) and decreasing in the seminiferous tubules with spermatocytes or later stages of spermatogenesis (Rajpert-De Meyts et al., 1999).

Although FSH is known to have a stimulating effect on Sertoli cell mitosis (Sharpe, 1994), it also contributes to the process of differentiation (Walker and Cheng, 2005) and synthesis of growth factors by Sertoli cells, which promote the maturation of Leydig cells (Sharpe, 1994; Walker and Cheng, 2005), making them able to respond to increasing LH stimuli and primarily synthesize testosterone.

Inactivation of AMH expression has been associated with the appearance of primary spermatocytes (Rajpert-De Meyts et al., 1999) and when androgen receptors reach adequate levels in Sertoli cells, AMH is inhibited, testosterone reaches the seminiferous tubules and spermatogonia enter meiosis (Al-Attar et al., 1997) and the expression of AMH can be used as a marker to determine the state of differentiation of Sertoli cells, as its expression drops sharply during the perinatal and prepubertal period (Al-Attar et al., 1997).

### Immunolocalization of AMH in fetal bovine ovaries

The immunolocalization of AMH in fetal ovaries showed a different pattern from that mentioned in the literature, with strong immunostaining in the cytoplasm of oocytes from primordial, primary, and secondary follicles and in granulosa cells in all fetal ages analyzed, contrary to what Vigier et al. (1984) mention that expression starts in the perinatal period. These results also differ from those observed in adult human ovaries, which show AMH restricted to granulosa cells of primary, secondary, preantral and antral follicles, not being present in GC of primordial follicles, oocyte, and ovarian stroma (Weenen et al., al., 2004). growing follicles. These differences may be due to physiological differences between species, but they may be due to variations in the methodology of the immunohistochemical technique.

Secondary and antral follicles were observed at 7 months showing a strong AMH labeling in the oocyte cytoplasm suggesting that, despite being produced by granulosa cells, this hormone is concentrated in the oocyte cytoplasm. The granulosa cells of these follicles also showed marking, although no marking was observed in the theca cells, which agrees with Münsterberg and Lovell-Badge (1991) who observed the expression of AMH in the granulosa cells of antral follicles and mention that theca cells do not express the AMH.

According to Nilsson et al. (2011), AMH inhibits the growth of primordial follicles and is in ovarian stromal cells that surround the nests of germ cells and neo-formed primordial follicles and in ovaries of adult animals, it inhibits the primordial transition to primary and is produced by granulosa cells of secondary follicles and in early antrum to antral formation. In this work we observed the presence of AMH in all ovarian follicles, from primordial to antral, both in the granulosa cells and in the oocyte cytoplasm, which agrees with what was observed in pigs by Almeida et al. (2012).

According to Dunlop and Anderson (2015), AMH is one of the most important factors in controlling the rate of activation of primordial follicles and plays a role in suppressing the recruitment of primordial follicles by reducing the expression of Kit Ligand and Fibroblast Growth Factor β (FGFβ) produced by the oocyte that seem to stimulate the transition from primordial to primary in several species however in this work we observed the presence of AMH in different phases of follicular growth suggesting other functions of this hormone in oocyte and follicular growth and also in ovarian development and, According to Modi et al. (2006), differences in AMH expression patterns between species make the understanding of AMH role in ovarian physiology difficult and considering that there are no studies in the literature on the presence of AMH in fetal bovine ovaries more studies are necessary for better understanding of the role of this protein in ovarian development.

In the ovary, AMH is expressed by preantral and small antral follicles (Weenen et al., 2004). AMH expression is detected in granulosa cells of activated primordial follicles but is absent in follicular stages following follicle-stimulating hormone (FSH)-dependent selection. Thus, AMH acts as a follicular gatekeeper ensuring that each small antral follicle (up to 8 mm) will produce little E2 prior to selection of the follicle that will undergo ovulation (Dewailly et al., 2014). AMH synthesized during the early ovarian follicular stages provides negative retrocontrol over folliculogenesis. It acts on two key mechanisms of folliculogenesis: first, on follicular recruitment: AMH has been shown to be a brake on the activation of primordial follicles, which prevents the recruitment of too large a follicle cohort in each menstrual cycle (La Marca and Volpe, 2006); secondly, on the stimulating effect of FSH on the follicles: AMH has a negative feedback on FSH and will decrease its level. The more follicles grow, the less AMH they synthesize. We therefore observe a decrease in this negative feedback, which allows mature follicles to be sensitive to FSH (Chang et al., 2013).

In pregnant buffaloes, the plasma AMH concentrations showed no correlation with AMH concentrations in their respective fetuses male or female, where the AMH concentration in males fetuses was 169-813 times greater than that in maternal circulation suggesting that AMH does not cross the placental barrier and AMH concentrations were significantly higher in male, with an increase according to fetal age, than in female fetuses. Also observed Immunolocalization of AMH buffaloes fetuses ovaries from the fifth month onwards and displayed intense immunostaining within the granulosa cells and in the cytoplasm of primordial follicle oocytes at 5, 6, 7, and 8 months (35–98cm CRL) and in the cytoplasm of pre-Sertoli cells within the sex cords (developing seminiferous tubules) at all ages (Costa et al., 2025).

Although the expression levels of AMH and AR in the ovary were lower compared to the testis, they still play roles in ovarian development and function and (Zhou et al., 2021; Liu et al., 2022). As an early marker of testicular differentiation, AMH is expressed in the embryonic testis but not the ovary in mammals, and its expression in the ovary starts after birth (37). In non-mammalian vertebrates such as birds and fish, AMH is expressed in the developing gonads of both sexes but predominantly in the testis. AMH expression in the early fetal testis is regulated by several key transcription factors, including SRY, SOX9, SF-1, WT1, and GATA4, suggesting an important role for AMH in male differentiation. (Cutting et al., 2014; Chen et al., 2017).

## Conclusion

In conclusion, Anti-Müllerian Hormone (AMH) is strongly expressed in Sertoli cells of testis and in granulosa cells of primordial, primary, secondary, and antral follicles of ovaries of bovine fetuses between 4 and 7 months, where the presence of AMH was also observed in the cytoplasm of the oocyte from these follicles, however further studies are needed to elucidate the molecular mechanisms and role of AMH in the development and gonadal physiology of bovine fetuses.

## Data Availability

Research data is only available upon request.
